# Impact of elevated pooling levels in basic medical insurance on insured individuals' inpatient service utilization—An equity analysis

**DOI:** 10.3389/fpubh.2026.1804493

**Published:** 2026-05-18

**Authors:** Chengrun Xie, Hongxi Wang, Jiaming Li, Jinxi Ding

**Affiliations:** School of International Pharmaceutical Business, China Pharmaceutical University, Nanjing, China

**Keywords:** basic medical insurance scheme for urban and rural residents, equity, hospitalization costs, inpatient service utilization, pooling model, provincial-level pooling

## Abstract

**Background:**

China has continuously promoted the transition of basic medical insurance from municipal-level pooling to provincial-level pooling to strengthen fund risk resilience, mutual-aid capacity, and benefit equalization. Although existing studies suggest that higher pooling levels may influence healthcare utilization, limited micro-level evidence exists on how provincial-level pooling of the Basic Medical Insurance Scheme for Urban and Rural Residents (BMISURR) affects inpatient service utilization, hospitalization costs, and income-related equity. This study examines the impact of provincial-level pooling on inpatient care among BMISURR enrollees from both utilization and equity perspectives.

**Methods:**

This study used seven waves of data from the China Family Panel Studies (CFPS) conducted between 2010 and 2022. After checking questionnaire skip patterns and excluding observations with missing key variables, the final analytical sample comprised 4,895 person-wave observations of BMISURR enrollees. A staggered difference-in-differences (DID) model with province and year fixed effects was employed to estimate the effects of provincial-level pooling on inpatient service utilization and hospitalization costs. An interaction specification was further used to compare the effects of two pooling models: the unified revenue-and-expenditure model and the provincial risk adjustment fund model. In addition, concentration indices (CI) and Wagstaff decomposition were applied to assess income-related inequality and identify the contribution of policy and socioeconomic factors to observed inequality in inpatient care.

**Results:**

Provincial-level pooling of BMISURR significantly increased both inpatient service utilization and hospitalization costs. The implementation of provincial-level pooling raised the probability of inpatient service utilization by 24.3% points and increased the average hospitalization costs per enrollee (including zeros for non-users) by approximately 445 yuan. Heterogeneity analysis by pooling model showed that both pooling models promoted inpatient utilization and expenditure, while the provincial risk adjustment fund model produced a stronger expansionary effect than the unified revenue-and-expenditure model. Concentration index (CI) results indicated that inpatient service utilization and hospitalization costs were concentrated among higher-income groups. Decomposition analysis further showed that income and healthcare resources were the dominant contributors to pro-rich inequality. Provincial-level pooling contributed to the pro-rich inequality pattern, accounting for 10.31% of inequality in inpatient service utilization and 12.78% in hospitalization costs. The interaction between pooling models contributed more substantially to inequality in hospitalization costs than to inequality in inpatient utilization, suggesting that institutional design differences are more closely related to expenditure disparities than to basic access disparities.

**Conclusion:**

Provincial-level pooling of BMISURR expanded inpatient service utilization and increased hospitalization costs among insured residents, with stronger effects observed under the provincial risk adjustment fund model. However, expanded pooling did not automatically translate into improved equity. Instead, inpatient care remained concentrated among higher-income groups, largely due to income stratification and unequal healthcare resource distribution. Future reforms should strengthen provincial fund governance, improve expenditure control under different pooling models, introduce targeted protections for low-income and medically vulnerable groups, promote more balanced healthcare resource allocation, and integrate provincial-level pooling with hierarchical diagnosis and treatment and with payment reform.

## Introduction

Elevating the pooling level of basic medical insurance is a central issue in the ongoing reform of China's medical security system. Following the rollout of landmark policy documents such as the Healthy China 2030 Plan Outline and the Opinions on Deepening the Reform of the Medical Security System, China has steadily advanced its basic medical insurance system, with notable progress in expanding population coverage, strengthening financing capacity, and improving benefit protection. Nevertheless, several structural challenges persist: the pooling level of medical insurance funds remains relatively low, the system's risk-sharing capacity is limited, and there are stark disparities in benefit levels and fund operation performance across regions (1). Against this backdrop, promoting the transition of basic medical insurance from municipal-level pooling to provincial-level pooling has emerged as a key institutional reform priority. Provincial-level pooling refers to higher-level integrated arrangements for basic medical insurance rules, fund management, and risk-sharing mechanisms across an entire provincial jurisdiction. Its core objective is threefold: first, to expand the size of the risk pool; second, to enhance the system's mutual aid capacity; and third, to narrow cross-prefectural disparities in benefit access and fund operation risks. These gaps arise from regional differences in fiscal capacity, demographic structure, and medical resource endowments, and provincial-level pooling addresses them by enforcing unified provincial constraints and governance rules (2–4). As intra-provincial population mobility expands and demand for cross-prefectural medical services grows, the fragmented municipal-level pooling model has become increasingly ill-equipped for addressing intra-provincial gaps in coverage equity and system sustainability. As such, provincial-level pooling constitutes far more than a mere administrative adjustment; it serves as a core institutional lever to enhance the equity and long-term resilience of China's basic medical insurance system.

This reform is especially critical for the Basic Medical Insurance Scheme for Urban and Rural Residents (BMISURR). Compared with employee-based medical insurance, BMISURR primarily covers non-employed and partially employed populations, including rural residents, older adults, students, and children. These groups are not only more vulnerable to health shocks, but also face multiple constraints in accessing healthcare: limited disposable income, higher non-medical care costs, weaker access to health information, and unequal access to higher-level medical institutions. In addition, BMISURR relies heavily on a financing structure composed of individual contributions and government subsidies. Compounding this structural feature, its insured population includes a high proportion of individuals with relatively limited earning capacity but comparatively high healthcare needs (5, 6). Official statistics released by the National Healthcare Security Administration (NHSA) illustrate this funding pressure: in 2023, the BMISURR fund recorded total revenues of 1,056.971 billion yuan and total expenditures of 1,045.765 billion yuan, leaving a minimal current surplus of 11.206 billion yuan. This narrow margin reflects a system that, despite its large overall scale, has very limited current reserve capacity (7). Meanwhile, the relatively high intensity of inpatient service utilization under BMISURR makes the system particularly sensitive to medical cost fluctuations and demographic pressure. Under the current municipal-level pooling framework, this pressure manifests as regionally divergent fund deficits and uneven benefit levels. Even economically developed prefecture-level regions have reported current deficits in BMISURR funds, forcing them to draw down accumulated balances to cover shortfalls. This indicates that municipal risk pools may be insufficient to absorb the impact of rising medical expenditures and demographic shocks. These strains are far more acute in regions facing rapid population aging, weaker local fiscal capacity, or mismatches healthcare resource allocations. This in turn further intensifies intra-provincial disparities in coverage equity and system sustainability (8, 9).

For this reason, the impacts of provincial-level pooling on BMISURR enrollees cannot be understood merely as an extension of standard insurance theory. Most conventional insurance models implicitly target urban working-age adults, who typically have stable earnings and reliable access to formal healthcare systems. By contrast, BMISURR enrollees typically make healthcare decisions under tighter budget constraints, heavier travel and caregiving burdens, and more unequal access to health information and care providers. Accordingly, for this population, the impacts of raising the pooling level depend not only on formal improvements in insurance benefits, but also on whether different subgroups can equally translate these institutional benefits into actual healthcare utilization. This population-specific perspective is particularly important when analyzing inpatient care, for two key reasons. First, hospitalization is usually the costliest and riskiest form of healthcare utilization, and it remains the core domain where basic medical insurance delivers its financial protection function (10, 11). Second, hospitalization decisions are highly sensitive to core insurance rules: reimbursement rates, deductibles, annual benefit ceilings, referral protocols, and the convenience of cross-regional settlement. As a result, changes to pooling arrangements tend to affect inpatient service utilization more directly than they affect routine outpatient care. They also more clearly reveal how institutional reform reshapes individual healthcare-seeking behavior and the distribution of household medical burdens.

Based on these characteristics, this study conceptualizes the impacts of provincial-level pooling on BMISURR enrollees through two interrelated channels: an incremental gain channel and a redistribution channel. The incremental gain channel refers to the expansion of effective coverage enabled by a higher pooling level. Provincial-level pooling can expand the overall risk pool, strengthen the system's risk-sharing capacity, and reduce the degree to which local fiscal capacity directly constrains benefit levels. In turn, this moves the system closer to the objective of provincial policy goal of “same system, similar benefits” (12, 13). For individual enrollees, these changes are reflected in multiple tangible improvements: higher reimbursement rates, adjusted deductibles and annual benefit ceilings, more unified benefit packages, broader reimbursement coverage, and more convenient intra-provincial settlement (14). These institutional adjustments can lower the effective financial threshold for hospitalization, and increase the probability that enrollees use inpatient services. At the same time, by expanding financial access to more treatments and reducing enrollees' out-of-pocket burdens, these reforms may also increase hospitalization costs.

However, provincial-level pooling may also function through a second, redistribution channel. This is because BMISURR enrollees vary dramatically along multiple dimensions: income, education, health literacy, mobility, caregiving resources, and proximity to high-quality care providers. As a result, they also differ in their ability to benefit from expanded coverage brought by pooling reform. Furthermore, under conditions of information asymmetry and professional dominance in healthcare provision, enhanced insurance payment capacity may not only release previously suppressed medical demand, but may also induce supply-side moral hazard, manifested as more intensive testing, overtreatment, and unnecessary hospitalization (15). Moreover, as cross-regional medical settlement becomes more convenient and provincial-level hospitals become more accessible, patients may be diverted from primary and county-level healthcare institutions to resource-rich higher-level hospitals across the province. In practice, these changes may disproportionately benefit socioeconomically advantaged individuals and residents of resource-rich regions, rather than disadvantaged sub groups. High-income and better-educated enrollees are typically better positioned to understand policy changes, afford transportation and caregiving costs, and access higher-quality care providers. By contrast, low-income groups, rural residents, and individuals facing greater non-medical access barriers may remain constrained in their access, even as nominal coverage expands. Under these conditions, provincial-level pooling may simultaneously increase overall inpatient utilization, while also driving a more unequal distribution of service utilization and hospitalization costs. This concern is particularly salient for BMISURR: the system has relatively limited financing growth potential, and its insured population is uniquely sensitive to both benefit expansion and unequal access to care. Without targeted supporting policies—including effective risk adjustment, hierarchical diagnosis and treatment mechanisms, and targeted protections for vulnerable groups, a higher pooling level may lead to accelerated upward referral flows and a greater concentration of fund payments among advantaged populations and regions (16).

Existing studies have primarily examined the impacts of health insurance on healthcare utilization, medical expenditures, and health outcomes across three core dimensions: insurance coverage, financing and benefit levels, and provider payment reform. Most studies show that health insurance participation significantly increases healthcare utilization and partially reduces household financial burden, while also generating incentives for service overuse and medical cost inflation (17–21). By contrast, the literature specifically focusing on the pooling level of basic medical insurance remains relatively scarce. Some studies analyze the impacts of provincial-level pooling on fund operating efficiency and risk-sharing capacity from an institutional perspective (22), while others examine its effects on fund revenue and expenditure or benefit levels using macro-level regional data (23). However, systematic micro-level evidence remains insufficient. In particular, there lack clear evidence on whether provincial-level pooling affects inpatient service utilization and hospitalization costs among BMISURR enrollees, and whether these reforms reduce or exacerbate access equity. Furthermore, existing studies also provide limited comparative evidence on whether different provincial-level pooling models—particularly the provincial unified revenue-and-expenditure model and the provincial risk adjustment fund model—generate different behavioral incentives and distributional outcomes.

Focusing on inpatient services is therefore especially critical when evaluating the impacts of raising the pooling level. Compared with outpatient visits or total healthcare utilization, inpatient service utilization and hospitalization costs have three distinctive features. First, inpatient care typically corresponds to severe or acute illness episodes, and is closely linked to household-level catastrophic health expenditure risk. This makes it the core domain in which medical insurance delivers its safety-net function (10, 11). Second, inpatient care relies heavily on hospital beds, specialized technology, skilled physicians, and hospital tiered hierarchy, and is deeply shaped by regional inequalities in healthcare resources; As a result, it accounts for the largest share of basic medical insurance fund expenditure (24, 25). Third, institutional changes associated with provincial-level pooling—including adjustments to benefit structure, reimbursement rules, ceiling changes, and settlement convenience—are likely to exert stronger marginal impacts on hospitalization decisions than on low-cost routine care. As such, examining inpatient service utilization and costs provides a particularly well-suited lens to observe how provincial-level pooling shapes high-cost medical behavior and how those impacts are distributed across socioeconomic groups.

Against this backdrop, this study takes China's provincial-level pooling reform for BMISURR as an institutional shock, and investigates its impact on enrollees' inpatient service utilization, hospitalization costs, and the distribution of these outcomes. Specifically, this paper addresses three research questions. First, it examines whether provincial-level pooling increases the probability that BMISURR enrollees use formal inpatient services, and whether it drives up average hospitalization costs. Second, it evaluates whether provincial-level pooling changes the income-related distribution of inpatient service utilization and hospitalization costs. Third, it compares the impacts of different provincial-level pooling models—specifically the provincial unified revenue-and-expenditure model and the provincial risk adjustment fund model—to identify whether differences in institutional design generate different behavioral and distributional patterns. By answering these questions, this study contributes to the existing literature in three key ways. First, it extends the literature on the pooling level of basic medical insurance by identifying micro-level causal impacts of provincial-level pooling on BMISURR enrollees' inpatient service utilization and hospitalization costs. Second, it integrates aggregate policy impacts and distributional patterns into a unified analytical framework, thereby enriching empirical research on the equity implications of medical insurance reform. Third, by comparing different provincial-level pooling models, it highlights the importance of institutional details and incentive structures in shaping policy outcomes. In turn, this provides more targeted empirical evidence for further optimizing BMISURR reform in China.

## Methods

### Policy context

Provincial-level pooling of basic medical insurance aims to enhance the system's risk resilience and mutual aid capacity by expanding the geographic scope of fund pooling and unifying revenue and expenditure management (9, 23). In China, the pooling level of basic medical insurance has been gradually upgraded from county-level or municipal-level to provincial-level. Drawing on official policy documents, this study summarizes the implementation timeline and institutional models of provincial-level pooling for the BMISURR, as shown in Table 1. The rollout of provincial-level pooling was highly staggered across provinces, and two distinct institutional models have emerged in practice.

**Table 1:** Current status of provincial-level pooling for BMISURR in China.

Province	Pooling time	Pooling model	
Tianjin	2010	Unified revenue and unified expenditure	
Chongqing	2011	Unified revenue and unified expenditure	
Hainan	2015	2015–2020	Risk adjustment fund
		2020–2022	Unified revenue and unified expenditure
Ningxia	2015	Risk adjustment fund	
Shanghai	2016	Unified revenue and unified expenditure	
Qinghai	2016	Risk adjustment fund	
Beijing	2018	Unified revenue and unified expenditure	
Xizang	2018	Unified revenue and unified expenditure	

In terms of institutional design, provincial-level pooling for BMISURR adopts follows typical models. The first is the “unified revenue and expenditure model”, under which the medical insurance fund is centrally managed at the provincial level, with uniform financing standards, benefit packages, and reimbursement policies across the entire province. The second is the “provincial risk adjustment fund model”, under which prefecture-level cities contribute a fixed share of their local fund revenue to a provincial-level adjustment fund. This fund is then redistributed to support regions facing fund deficits. Notably, some provinces that adopted the risk adjustment fund model have not fully unified local benefit policies. To ensure valid causal identification and comparable institutional impacts, this study only includes provinces that achieved full policy uniformity and improved benefit levels after pooling reform. These sample provinces in this study are Tianjin, Chongqing, Hainan, Ningxia, Shanghai, Qinghai, Beijing, and Xizang. Notably, Hainan implemented the risk adjustment fund model during 2015–2020, and switched to the unified revenue-and-expenditure model from 2020 onward. All other sample provinces maintained a single pooling model throughout the entire study period. The staggered implementation timing and distinct institutional models provide a credible quasi-experimental setting, which supports the staggered difference-in-differences (DID) estimation strategy adopted in this study.

### Data sources and audited sample construction

This study uses seven waves of the China Family Panel Studies (CFPS, 2010–2022), a national representative longitudinal household survey conducted by the Institute of Social Science Survey at Peking University. Two analytical files are constructed: a harmonized person—wave CFPS panel (62,062 observations) for questionnaire logic verification, and a restricted BMISURR analytical extract (4,970 observations) for econometric estimation. The harmonized panel is used to validate skip patterns for inpatient service utilization and hospitalization cost, while the BMISURR extract serves as the core estimation sample.

A key data audit confirms that hospitalization cost in the CFPS is a conditional survey item: it is only asked of respondents who reported inpatient utilization in the past 12 months. Thus, “not applicable” values for non-hospitalized respondents reflect structural non-applicability rather than random missingness. In this audited analysis, hospitalization cost is coded as 0 for respondents with no inpatient use. Only observations with genuine missing hospitalization cost among hospitalized respondents are treated as missing outcome data.

The sample is restricted to enrollees in the BMISURR and exclude observations with incomplete key variables: 1 with unknown inpatient utilization, 10 with genuine missing hospitalization cost among hospitalized respondents, 56 with missing hukou status, 2 with missing marital status, 2 with missing chronic disease status, and 4 with missing provincial-level healthcare resource data. The final audited analytical sample consists of 4,895 person-wave observations, covering provinces that implemented provincial-level pooling of BMISURR during 2010–2022.

### Variable selection

#### Dependent variables

This study employs two core dependent variables consistent with standard health economics research conventions. (1) Inpatient service utilization: A binary indicator measured by the CFPS question “Have you been hospitalized due to illness in the past 12 months?”, coded 1 for “Yes” and 0 for “No”. (2) Hospitalization costs (yuan): “In the past 12 months, including reimbursed and expected reimbursable portions, how much did your hospitalization cost in total?”.

#### Independent variables

Two key independent variables are defined to identify the policy impacts and model heterogeneity, aligned with the staggered DID framework and interaction term specification. (1) Provincial-level pooling (DID): A binary indicator for the implementation of provincial-level pooling of BMISURR. Official policy documents are manually collected from provincial healthcare security bureaus to identify the actual implementation year (rather than the policy issuance year) of provincial-level pooling. The variable equals 1 if the province has implemented provincial-level pooling before or during the survey year, and 0 otherwise. (2) Pooling model (Mode): A time-invariant binary indicator for the institutional model of provincial-level pooling. It is coded 1 for provinces adopting the “provincial risk adjustment fund model”, and 0 for those adopting the “unified revenue-and-expenditure model”. This single indicator is used to construct the interaction term DIDit × Modei, which directly tests the differential impacts between the two models.

#### Control variables

Drawing on Grossman's health demand model (26) and to address confounding reforms (per reviewer comment 3), A comprehensive set of individual, household, and provincial-level control variables is included: (1) Individual characteristics: Age (continuous); Gender (1 = male, 0 = female); Marital status (1 = married/cohabiting, 0 = unmarried/divorced/widowed); Educational attainment (0 = illiterate/semi-literate, 1 = primary school, 2 = junior high school, 3 = senior high/vocational school, 4 = college and above); Household registration (1 = rural, 0 = urban); Income (natural logarithm of personal annual income plus 1). (2) Health behaviors and status: Smoking (1 = yes, 0 = no); Drinking (1 = yes, 0 = no); Health status (1 = unhealthy, 2 = fairly healthy, 3 = healthy, aggregated from the original 5-point CFPS scale). (3) Provincial-level characteristics: Economic development level (natural logarithm of per capita GDP); Healthcare resources (number of hospitals per 10,000 persons by province).

### Statistical model

#### Staggered difference-in-differences model

Using the staggered rollout of provincial-level pooling for BMISURR across provinces, this study adopts a staggered difference-in-differences (DID) design with two-way fixed effects to estimate the causal impact of provincial-level pooling on inpatient service utilization and hospitalization costs. The baseline model is specified as:


$$\begin{array}{l} Y_{ipt}=\beta_{0}+\beta_{1}Pooling_{pt}+\gamma X_{ipt}+{\text{λ}}_{p}+\delta_{t}+\varepsilon_{ipt} \end{array}$$ (1)

Subscripts *i*, *p*, and *t* denote individual, region, and time, respectively. The dependent variable *Y*_*ipt*_represents the inpatient service utilization or hospitalization costs of individual *i* in province *p* during period *t*. The core explanatory variable *Pooling*_*pt*_indicates whether province *p* had implemented provincial pooling of BMISURR during period *t*. If provincial-level pooling had been implemented, *Pooling*_*pt*_ = 1; otherwise, *Pooling*_*pt*_= 0. In this setting, provinces that have implemented provincial-level pooling constitute the experimental group, while those that have not form the control group, *X*_*ipt*_ denotes the full set of control variables; λ_*p*_ represents the province fixed effect; δ_*t*_ represents the year fixed effect; ε_*ipt*_ is the random disturbance term.

#### Interaction model for pooling model heterogeneity

To validly compare the differential impacts between the two provincial-level pooling models, an interaction specification is estimated, in which the interaction between the pooling policy and the model type is included, thereby replacing the less reliable split-sample comparison. The model is:


$$\begin{array}{l} Y_{ipt}=\beta_{0}+\beta_{1}Pooling_{pt}+\beta_{2}\left(Pooling_{pt}\times Model_{p}\right)+\gamma X_{ipt}+{\text{λ}}_{p}\\ \;+\delta_{t}+\varepsilon_{ipt} \end{array}$$ (2)

Where *Model*_*p*_ is a time-invariant indicator equal to 1 for provinces adopting the “provincial risk adjustment fund model”, and 0 for those adopting the “unified revenue-and-expenditure model”. β_1_ captures the policy impact of the unified revenue-and-expenditure model. β_2_ is the coefficient of interest, representing the differential impact of the risk adjustment fund model relative to the unified model. The total impact of the risk adjustment fund model is β_1_ + β_2_, which is tested using the lincom command. For model heterogeneity analysis, separate split-sample regressions are no longer used as in the earlier version of the manuscript. Instead, an interaction model is estimated within the full analytical framework. Notably, because Hainan switched from the risk adjustment fund model to the unified revenue-and-expenditure model in 2020, it is excluded from the model heterogeneity analysis to avoid classification ambiguity.

### Concentration index decomposition

To analyze income-related equity in inpatient service utilization and costs, this study employs the Concentration Index (CI) and the Wagstaff decomposition method (27). The CI is a standard metric for quantifying socioeconomic inequality in health and healthcare utilization. Its key advantages include cross-population comparability, invariance to the outcome mean, and the ability to identify contributing factors via decomposition (28–34). The CI is calculated as:


$$\begin{array}{l} CI=2cov\left(\;y_{i\;},R_{i}\right)/\mu \end{array}$$ (3)

Where *y*_*i*_ represents the outcome variable of service utilization, μ denotes the population mean of this variable, and *R*_*i*_ indicates the score rank of sample *i* in the income distribution. The *CI* ranges from (−1, 1). *CI* > 0 indicates pro-rich inequality in the outcome variable, while a *CI* < 0 indicates pro-poor inequality. A larger absolute value of *CI* signifies that the distribution of the outcome variable is more sensitive to income levels, reflecting a greater degree of inequity.

This study employs the Centralized Index Decomposition Method proposed by Wagstaff (35) to decompose factors that potentially affect the equity of inpatient services for insured individuals. By ranking the contribution of different decomposed factors to overall inequity, the primary sources of inequity are identified, which can inform targeted policy interventions.

The decomposition formula is as follows:


$$\begin{array}{l} C=\underset{j}{\sum }\left(\beta_{j}{\bar{X}}_{j}/\mu \times {CI}_{j}\right)+GC_{\varepsilon }/\mu \end{array}$$ (4)

*C* is the non-standardized concentration index. β_*j*_ is the marginal effect from a linear regression of *y*_*i*_ on determinant *j*. $\beta_{j}{\bar{X}}_{j}/\mu \times {CI}_{j}$ denotes the contribution of influencing factor *j* to inequality in service utilization. *GC*_ε_ is the concentration index of the residual term. μ is the mean of the outcome variable. In this decomposition, provincial pooling status and pooling model type are explicitly included as core policy determinants, allowing us to estimate their individual contributions to inequity in inpatient care.

### Statistical analysis

All analyses were conducted in Stata 18.0. The characteristics of the final analytical sample were first described using summary statistics for all study variables. Continuous variables were presented as means with standard deviations, while categorical variables were summarized as frequency proportions. Based on the audited CFPS questionnaire logic, hospitalization cost was coded as 0 for respondents without inpatient service utilization, whereas only genuine missing values among hospitalized respondents were treated as missing outcome data.

The empirical analysis then proceeded in three stages. First, the overall association between provincial-level pooling of BMISURR and the two study outcomes—inpatient service utilization and hospitalization costs—was estimated, after adjustment for individual- and province-level covariates as well as province and year fixed effects. Second, it is examined whether these associations differed by pooling model, by comparing the impacts of the unified revenue-and-expenditure model and the provincial risk adjustment fund model. Third, income-related inequality in both outcomes was evaluated, with the contribution of policy and socioeconomic factors to observed inequality patterns being quantified.

To assess the robustness of the main findings, a series of supplementary analyses was conducted, including pre-trend tests, placebo tests based on random reassignment of treatment timing, adjustment for concurrent healthcare reforms, propensity score matching combined with difference-in-differences estimation, and heterogeneity-robust estimators for staggered policy adoption. Standard errors were clustered at the province level in all regression models. All statistical tests were two-sided, and *p*-values below 0.05 were considered statistically significant.

## Results

### Sample construction and missing-data audit

**Figure 1:**
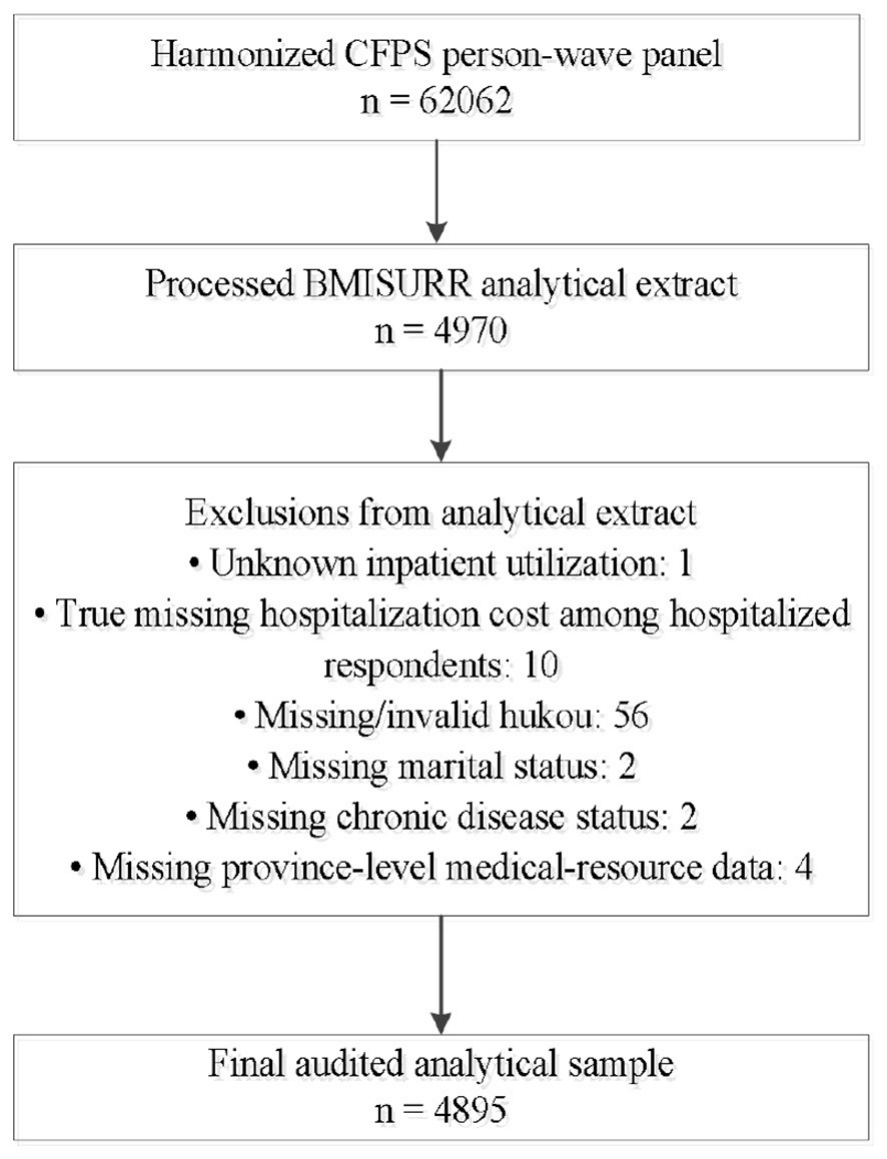
Audited sample construction after correcting the hospitalization-cost skip pattern.

Figure 1 summarizes the audited sample construction. The essential correction concerns hospitalization cost: the large mass of “not applicable” values among respondents without inpatient service utilization reflected structural non-applicability rather than genuine missingness. After the correction, only 10 observations with genuine missing hospitalization cost among hospitalized respondents remained.

Table 2 reports the audited sample counts. In the retention test, exclusion from the final analytical sample was not significantly associated with the treatment indicator (β = 0.010, *SE* = 0.018, *p* = 0.565). This suggests that exclusion within the processed BMISURR analytical extract was not systematically driven by provincial-level pooling status.

**Table 2:** Audited sample construction.

Step	Observations
Processed BMISURR analytical extract	4,970
Exclude: unknown inpatient utilization	1
Exclude: true missing hospitalization cost among hospitalized respondents	10
Exclude: missing/invalid hukou	56
Exclude: missing marital status	2
Exclude: missing chronic-disease status	2
Exclude: missing province-level healthcare-resource data	4
Final audited analytical sample	4,895

Table 3 compares retained and excluded observations within the processed BMISURR analytical extract. Excluded observations were younger, more urban-skewed, and somewhat more likely to be in treated province-years, but the direct retention model test not identify a statistically significant association between exclusion and the treatment indicator.

**Table 3:** Comparison of retained and excluded observations within the processed BMISURR analytical extract.

Variable	Retained	Excluded	Difference
Age	48.842	43.507	5.335
Male	0.442	0.453	−0.011
Educational attainment	1.715	2.347	−0.632
Household registration	0.498	0.08	0.418
Marital status	0.888	0.493	0.395
Income	6.503	7.948	−1.445
Health status	2.221	2.64	−0.419
Smoking	0.269	0.32	−0.051
Drinking	0.134	0.08	0.054
Treatment	0.05	0.107	−0.057

### Basic profile of respondents

Table 4 reports descriptive statistics for the final audited analytical sample. The mean inpatient service utilization rate was 32.3%, and mean hospitalization cost for the full sample—including zero costs among non-hospitalized respondents—was 1,707.5 yuan. The average respondent was 48.8 years old, 44.2% were male, and the mean self-rated health score was 2.22 on the harmonized CFPS scale.

**Table 4:** Descriptive statistics of variables.

Variable	Mean	*SD*	Min	Max
Inpatient service utilization	0.323	0.350	0	1
Hospitalization costs	1,707.468	10,905.420	0	400,000
Pooling	0.037	0.189	0	1
Age	48.842	11.853	18	88
Gender	0.442	0.497	0	1
Educational attainment	1.715	1.304	0	4
Household registration	1.498	0.500	1	2
Marital status	0.888	0.316	0	1
Income	6.503	4.731	0.000	13.017
Health status	2.221	1.079	1	5
Smoking	0.269	0.443	0	1
Drinking	0.134	0.341	0	1
Economic level	10.765	0.468	9.882	12.155
Healthcare resources	0.211	0.076	0.026	0.407

### Impact of implementing provincial-level pooling of basic medical insurance on hospitalization service costs

The results of the baseline regression examining the impact of provincial-level pooling of basic medical insurance on insured individuals' inpatient service utilization and hospitalization costs are presented in Table 5. The implementation of the provincial-level pooling policy has significant and positive impacts on both insured individuals' inpatient service utilization and their hospitalization costs, with policy variable coefficients of 0.243 and 445.138, respectively. In terms of economic significance, the introduction of provincial-level pooling increases the probability of inpatient service utilization by approximately 24.3 percentage points and raises hospitalization costs by about 445 yuan per person. These findings indicate that provincial-level pooling of basic medical insurance not only increases insured individuals' utilization of inpatient services but also leads to a substantial rise in their hospitalization expenses.

**Table 5:** Baseline regression results.

Variable	Inpatient service utilization	Hospitalization costs
Pooling	0.243^***^	445.138^***^
	(0.013)	(117.261)
Age	0.001^**^	2.669
	(0.000)	(2.536)
Gender	0.016	331.388^***^
	(0.011)	(81.166)
Educational attainment	0.001	11.333
	(0.005)	(25.360)
Household registration	0.031^*^	73.135
	(0.015)	(50.917)
Marital status	0.017	45.860
	(0.018)	(140.331)
Income	−0.000	2.999
	(0.001)	(5.827)
Health status	−0.043^***^	−190.599^***^
	(0.004)	(43.096)
Smoking	−0.028^**^	−219.586^**^
	(0.011)	(81.008)
Drinking	−0.010	−204.610^**^
	(0.014)	(78.955)
Economic level	0.085	24.412
	(0.056)	(278.482)
Healthcare resources	0.260^*^	1,059.756
	(0.139)	(1,086.070)
Province FE	Yes	Yes
Year FE	Yes	Yes
*N*	4,895	4,895
*R* ^2^	0.052	0.026

Robust standard errors are shown in parentheses. ^*^p < 0.10, ^**^p < 0.05, ^***^p < 0.01.

### Impacts of different provincial-level pooling models for basic medical insurance on hospitalization services and costs

Heterogeneity analysis is conducted to compare the differential policy impacts of the two dominant provincial-level pooling models. To avoid bias from less reliable split-sample comparisons, this study adopts an interaction specification that incorporates the interaction term of the pooling policy indicator and the model type dummy (Table 6).

**Table 6:** Heterogeneity analysis of pooling models.

Variable	Inpatient service utilization	Hospitalization costs
Pooling × model	0.054^***^	31.403^**^
	(0.012)	(15.001)
Pooling	0.213^***^	333.361^**^
	(0.017)	(133.712)
Controls	Yes	Yes
Province FE	Yes	Yes
Year FE	Yes	Yes
*N*	4,464	4,464
*R* ^2^	0.050	0.026

The heterogeneity analysis is estimated on a restricted sample excluding Hainan, because Hainan switched from the risk adjustment fund model to the unified revenue-and-expenditure model in 2020. This avoids ambiguity in the time-invariant classification of pooling model, and ensures comparability across model types. ** indicates p < 0.05, and *** indicates p < 0.01.

The time-invariant dummy variable Model distinguishes between the two institutional designs: it equals 1 for provinces implementing the provincial risk adjustment fund model, and 0 for those adopting the unified revenue-and-expenditure model. The coefficient of the interaction term Pooling × Model captures the incremental impact of the risk adjustment fund model relative to the unified model, while the main effect of Pooling represents the standalone policy impact of the unified revenue-and-expenditure model.

Regression results confirm significant heterogeneous impacts across the two models. The interaction term is statistically significant for both inpatient service utilization and hospitalization costs, indicating that the risk adjustment fund model exerts a substantially stronger positive effect on both outcomes than the unified revenue-and-expenditure model.

### Impacts of provincial-level pooling of basic medical insurance on the equity of inpatient services for insured persons

#### Equity in inpatient service utilization

Both the implementation of provincial-level pooling of basic medical insurance and different provincial-level pooling models exert heterogeneous impacts on insured individuals' inpatient service utilization and hospitalization costs. Therefore, it is critical to further examine the impacts of provincial-level pooling on the equity of inpatient service utilization and costs from the perspective of the insured population.

To investigate how the implementation of provincial-level pooling and different provincial-level pooling models affect the equity of inpatient service utilization and hospitalization costs among insured individuals, this study first applies the concentration index to assess the equity of inpatient service utilization and costs, and then further analyzes the impacts of provincial-level pooling on equity via concentration index decomposition.

**Figure 2:**
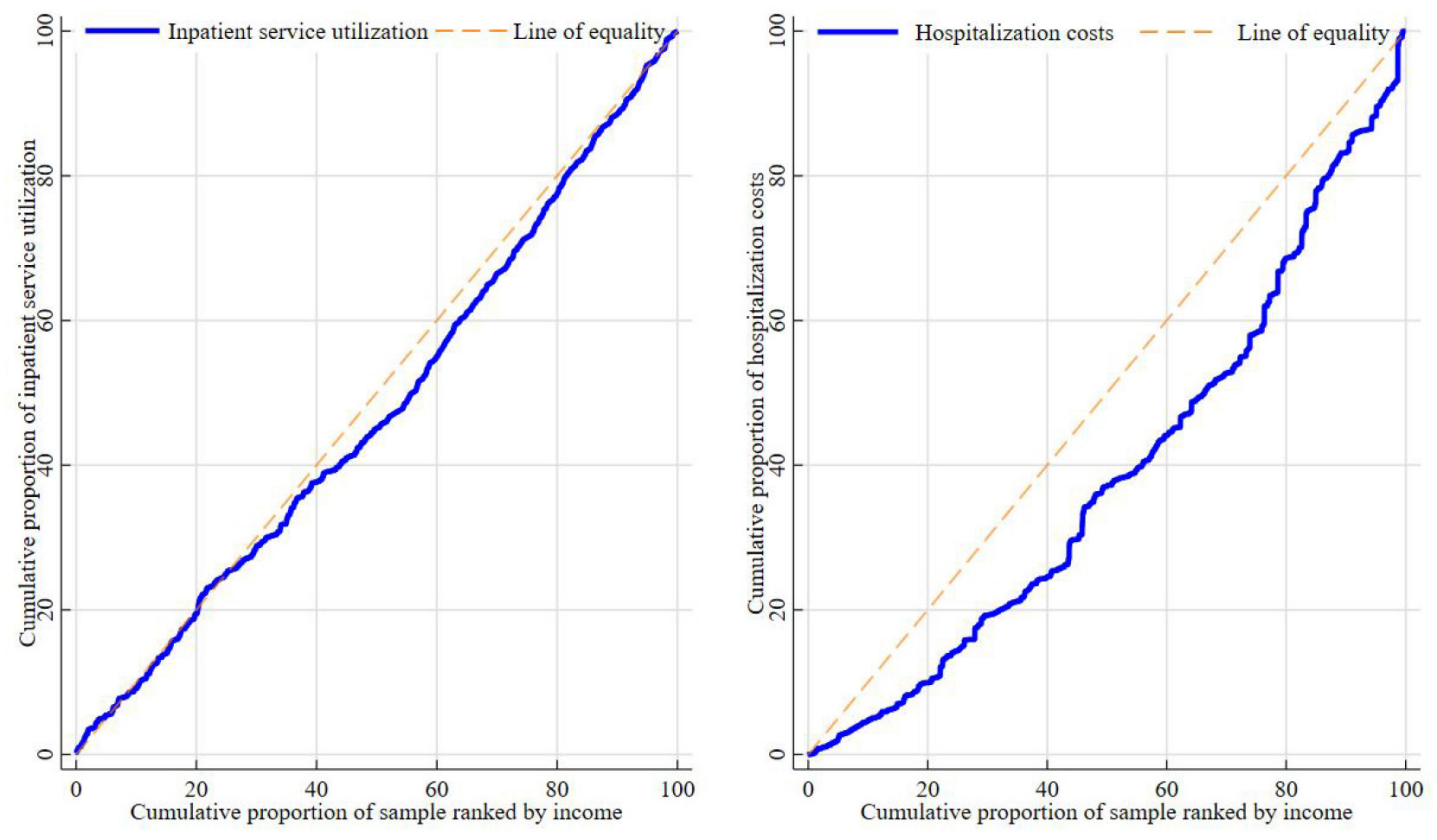
Equity analysis results.

In terms of equity, the concentration indices (CI) for inpatient service utilization and hospitalization costs are 0.029 and 0.214, respectively. Both CIs are positive and statistically significant (Table 7). The concentration curves are concave and lie below the line of absolute equity, indicating that inpatient service utilization and hospitalization costs are distributed in favor of higher-income groups (Figure 2).

**Table 7:** Concentration index test results for equity in inpatient services for insured individuals.

Project	Method	95% *CI*	Standard error	Significance
Inpatient services utilization	Delta	0.011–0.047	0.011	< 0.001^***^
	Bootstrap	0.013–0.045	0.012	< 0.001^***^
Hospitalization costs	Delta	0.102–0.326	0.042	< 0.001^***^
	Bootstrap	0.111–0.317	0.052	< 0.001^***^

The number of samples for Bootstrap method verification is 1,000; ^***^indicates significance at the 1% significance level.

### Impacts of provincial-level pooling policy on equity of inpatient services for insured individuals

Taking the implementation of provincial-level medical insurance pooling as the core explanatory variable and insured individuals' inpatient service utilization and hospitalization costs as the outcome variables, this study further decomposes the concentration index to identify the relative contribution of policy, socioeconomic, health, and healthcare supply factors to income-related inequality in inpatient care. The decomposition results are shown in Tables 8, 9.

**Table 8:** Decomposition results of the concentration index for inpatient service utilization.

Variables	Modulus of elasticity	Concentration index	Contribution level	Contribution rate (%)
Pooling	0.042	0.069	0.003	10.31
Age	0.050	−0.033	−0.0017	−5.84
Gender	−0.045	0.144	−0.0065	−22.34
Educational attainment	−0.015	0.230	−0.0035	−12.02
Household registration	0.060	0.065	0.0039	13.40
Marital status	0.040	−0.013	−0.0005	−1.73
Income	0.057	0.456	0.026	89.35
Health status	−0.120	0.075	−0.0090	−30.93
Smoking	−0.010	0.092	−0.0009	−3.10
Drinking	−0.005	0.088	−0.0004	−1.38
Economic level	−0.080	0.012	−0.0010	−3.43
Healthcare resources	0.079	0.289	0.023	79.04

**Table 9:** Decomposition results of the concentration index for hospitalization costs.

Variables	Modulus of elasticity	Concentration index	Contribution level	Contribution rate (%)
Pooling	0.3820	0.0715	0.0273	12.78
Age	−0.2915	−0.0350	0.0102	4.77
Gender	0.3964	0.1480	0.0587	27.44
Educational attainment	0.0184	0.2281	0.0042	1.96
Household registration	0.2411	0.0663	0.0160	7.48
Marital status	0.1327	−0.0131	−0.0017	−0.80
Income	0.3411	0.4661	0.1590	74.33
Health status	−1.0842	0.0761	−0.0825	−38.57
Smoking	−0.1653	0.0938	−0.0155	−7.25
Drinking	−0.0824	0.0895	−0.0074	−3.45
Economic level	5.9823	0.0128	0.0766	35.81
Healthcare resources	0.3985	0.1129	0.0450	21.02

The results indicate that income is the most significant positive contributor to pro-rich inequality in both inpatient service utilization and hospitalization costs. Specifically, income accounts for 89.35% of the observed inequality in inpatient service utilization and 74.33% of the inequality in hospitalization costs. This finding suggests that socioeconomic stratification remains the dominant underlying force shaping inpatient inequality: even under the same insurance system, higher-income individuals are better able to afford deductibles and non-medical costs, access policy information, and utilize higher-level inpatient services. In addition, healthcare resources are another major contributor to inequality, accounting for 79.04% of the inequality in inpatient service utilization and 21.02% of the inequality in hospitalization costs. This implies that the uneven distribution of hospitals and medical capacity across regions constitutes a crucial supply-side pathway through which income-related inequality is reproduced. In better-resourced areas, insured individuals tend to obtain timely admission and access higher-quality inpatient care, which disproportionately benefits populations with greater economic means, mobility, and information access.

By comparison, the contribution of the provincial-level pooling policy itself exerts a positive and policy-relevant impact, yet its independent contribution is weaker than those of income and healthcare resources. Provincial-level pooling contributes 10.31% to the observed inequality in inpatient service utilization and 12.78% to the observed inequality in hospitalization costs. These findings suggest that provincial-level pooling should be interpreted as one factor associated with a broader structure of socioeconomic and regional inequality, rather than as the sole source of inequity in inpatient care. It should also be noted that, in concentration index decomposition, the sum of contribution rates may exceed 100%. This is mathematically possible when certain covariates make strong positive contributions to pro-rich inequality while other variables exert offsetting negative contributions. In the present analysis, factors such as health status and gender partially offset the inequality driven by income gaps and healthcare resource allocation. This offset mechanism clarifies explains why the relative contribution of dominant positive factors can be very large. Therefore, contribution rates exceeding 100% do not imply that a single variable literally explains more than total inequality; rather, they indicate that this factor is the dominant positive contributor in the presence of countervailing effects from other determinants. Furthermore, the decomposition results show that provincial-level pooling contributes more to the observed inequality in hospitalization costs than in inpatient service utilization, indicating a stronger association with disparities in financial burden than with disparities in basic service use. These decomposition results should be interpreted as partitioning the observed income-related inequality across covariates, rather than as demonstrating that provincial pooling causally changed the level of inequality.

### Impacts of provincial-level pooling model on equity of inpatient services for insured individuals

To further examine how institutional design shapes equity outcomes, this study decomposes the concentration index across different pooling models. The results are presented in Tables 10, 11.

**Table 10:** Concentration index decomposition of inpatient service utilization.

Variables	Modulus of elasticity	Concentration index	Contribution level	Contribution rate (%)
Pooling × model	0.0019	0.5081	0.0010	3.45
Age	0.1012	−0.0333	−0.0034	−1.15
Gender	0.0040	0.1431	0.0006	1.98
Educational attainment	−0.0020	0.2302	−0.0005	−1.59
Household registration	0.0050	0.0652	0.0003	1.12
Marital status	0.0100	−0.0126	−0.0001	−0.44
Income	0.0536	0.3809	0.0204	70.23
Health status	−0.1570	0.0752	−0.0118	−40.55
Smoking	−0.0050	0.0924	−0.0005	−1.59
Drinking	−0.0050	0.0887	−0.0004	−1.53
Economic level	−0.1000	0.0125	−0.0013	−4.31
Healthcare resources	0.1170	0.0617	0.0072	24.74

**Table 11:** Concentration index decomposition of hospitalization costs.

Variables	Modulus of elasticity	Concentration index	Contribution level	Contribution rate (%)
Pooling × model	0.1567	0.5081	0.0796	37.21
Age	−0.3270	−0.0333	0.0109	5.09
Gender	0.3125	0.1441	0.0450	21.04
Educational attainment	0.0225	0.2302	0.0052	2.42
Household registration	0.2696	0.0649	0.0175	8.18
Marital status	0.1460	−0.0126	−0.0018	−0.86
Income	0.2369	0.3809	0.0902	42.19
Health status	−0.8620	0.0749	−0.0646	−30.20
Smoking	−0.1831	0.0924	−0.0169	−7.91
Drinking	−0.0923	0.0883	−0.0081	−3.81
Economic level	−0.8130	0.0123	−0.0100	−4.67
Healthcare resources	1.1402	0.0617	0.0704	32.89

The decomposition confirms that income remains the dominant structural driver of pro-rich inequality across model-specific specifications. In the pooling-model analysis, income contributes 70.23% of inequality in inpatient service utilization and 42.19% of inequality in hospitalization costs. When combined with the baseline decomposition results, this finding underscores that the core inequality in inpatient care is rooted in differences in economic capability rather than in policy design alone. At the same time, healthcare resources continue to play a major role, contributing 24.74% of inequality in inpatient service utilization and 32.89% of inequality in hospitalization costs. This highlights the importance of healthcare supply conditions in shaping the distribution of the gains from insurance reform. In contexts where higher-quality inpatient services are concentrated in better-resourced regions, any policy that expands household purchasing power may disproportionately benefit individuals and groups who are already better positioned to access those resources.

Against this broader structural backdrop, the contribution of the Pooling × Model term remains substantively important, particularly for hospitalization costs. The interaction term contributes 3.45% of the inequality in inpatient service utilization but 37.21% of the inequality in hospitalization costs. This indicates that the provincial-level risk adjustment fund model is more strongly associated with disparities in financial burden than with disparities in basic inpatient access. This suggests that differences in pooling design shape how existing structural inequalities are reflected in observed expenditure inequality. Consistent with the baseline decomposition analysis, some variables yield negative contribution values. In particular, health status contributes −40.55% to inequality in inpatient service utilization and −30.20% to inequality in hospitalization costs. This indicates that poorer health status is disproportionately concentrated among lower-income individuals. By increasing medical need among disadvantaged groups, this factor partially offsets the pro-rich inequality observed in the sample. This also explains why some positive contribution rates appear unusually large in relative terms, as they partially offset the negative contributions from other factors. Overall, the decomposition results suggest that the observed inequality in inpatient care stems from the combined effects of income stratification, unequal healthcare resource distribution, and institutional differences in pooling design. Among these, income and healthcare resources represent the dominant structural drivers. In contrast, provincial-level pooling and pooling-model differences are associated with the observed inequality pattern alongside these deeper structural factors, rather than independently generating causal inequality effects.

### Robustness checks

#### Parallel trends test

**Figure 3:**
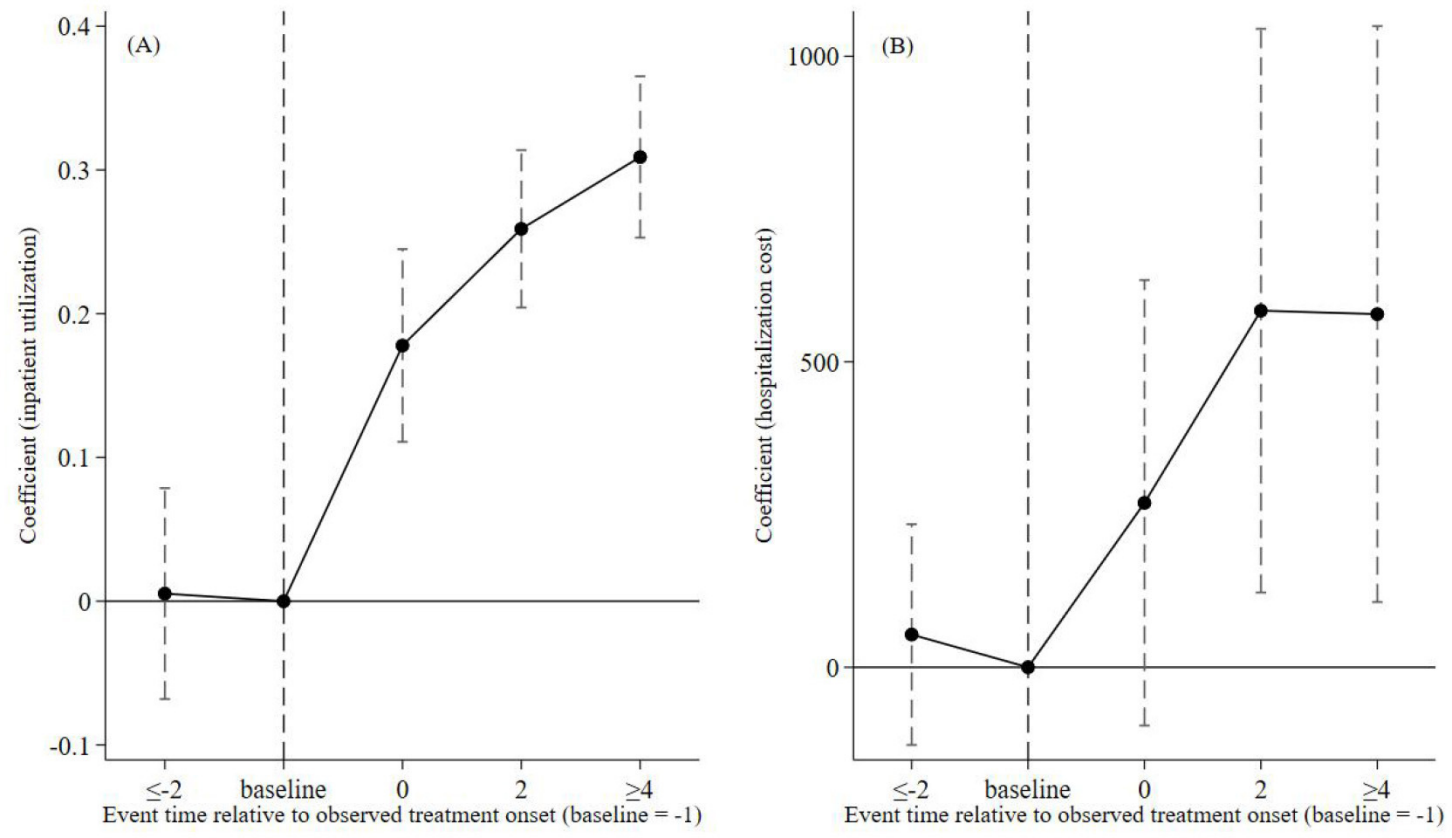
Parallel trend test.

The parallel trends and dynamic effects are shown in Figures 3A, B. All pre-treatment estimated coefficients are statistically insignificant prior to the reform. Although the hospitalization cost outcome shows minor non-parallel fluctuations in the earlier lead period. The event-study evidence is therefore interpreted with caution and the full set of robustness strategies is adopted—including placebo tests, PSM-DID, and heterogeneity-robust estimators—to verify the validity of causal identification. After implementation (event time ≥ 0), coefficients for inpatient service utilization turn positive and remain significantly positive, while coefficients for hospitalization costs also stay above zero. These patterns confirm a sustained and significant policy effect.

#### Placebo test

**Figure 4:**
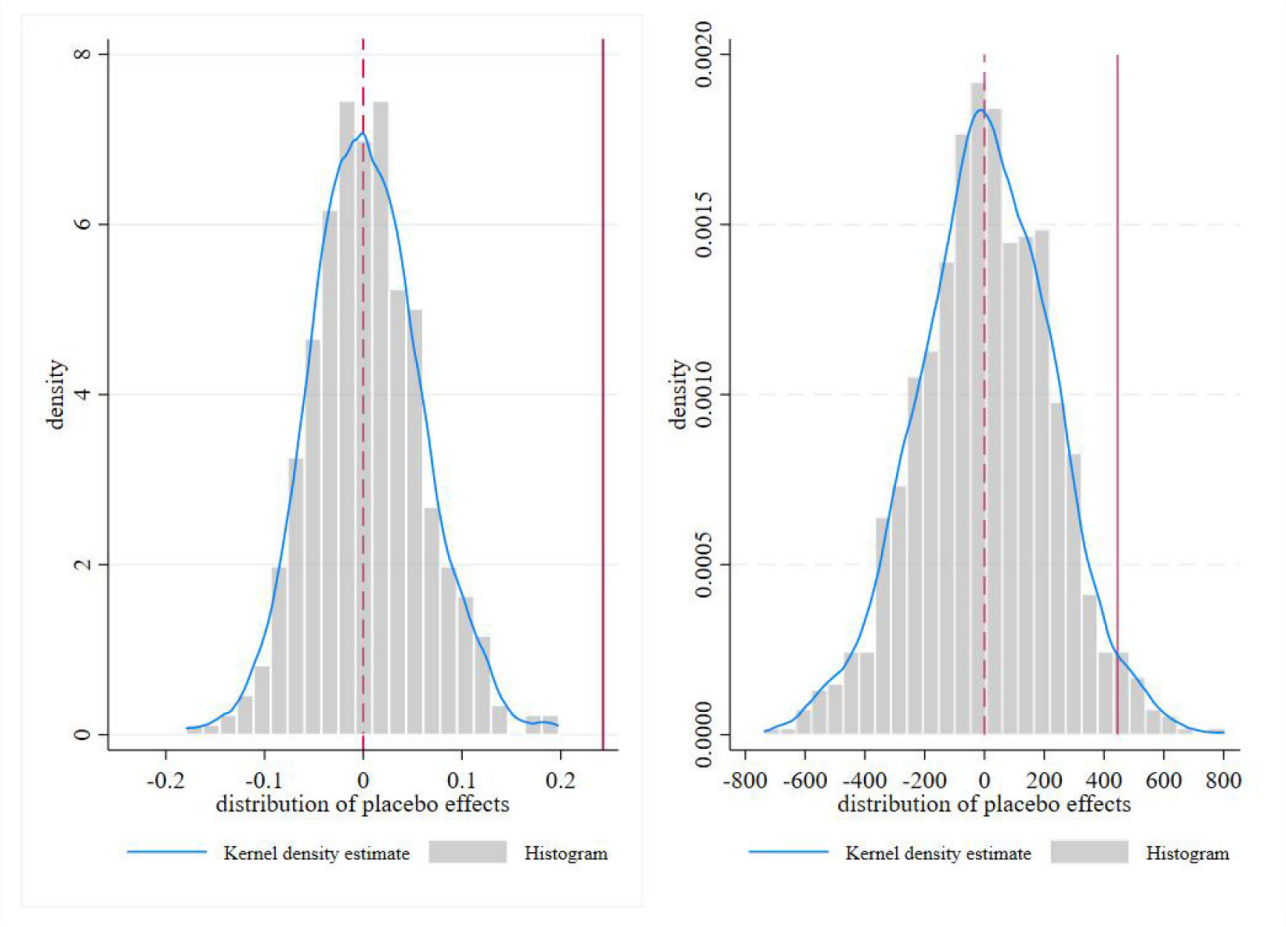
Placebo test.

Placebo tests are conducted to rule out the possibility that our baseline DID estimates for inpatient service utilization and hospitalization costs were driven by unobserved confounding factors or random chance (Figure 4). As illustrated in the figures, the distribution of coefficients generated from 500 random assignments of pseudo-policy shocks is centered around zero, consistent with the null hypothesis of no systematic effect. In contrast, the genuine treatment effect estimates lie far in the right tail of these placebo distributions, clearly separated from the bulk of simulated coefficients. This evidence confirms that our main findings are not artifacts of unaccounted trends or random noise, but reflect the causal impact of the pooling reform on healthcare utilization and spending.

#### Controlling for concurrent policies

To address potential confounding bias from concurrent healthcare reforms, additional control is introduced for three major policy shocks (Table 12). First, the study accounts for provincial centralized drug procurement (drug_procure), which substantially reduced inpatient drug expenditures over the sample period. Its aggregate trend is absorbed by year fixed effects, while staggered regional rollout is captured by province-year dummies so as to isolate the cost-containment effect. Second, the study control for payment system reform (pay_reform) using a province-year dummy based on the actual timing of DRG/DIP adoption. Third, the study incorporate grassroots medical capacity (grassroot_cap), measured by the number of general practitioners per 10,000 population, to capture the diversionary effect of strengthened primary care on inpatient patterns. All three policies exhibit clear cross-provincial and intertemporal heterogeneity. After controlling for these confounding factors, our core baseline findings remain fully consistent and robust.

**Table 12:** Controlling for concurrent policies.

Variable	Inpatient service utilization	Hospitalization costs
Pooling	0.218^***^	386.472^***^
	(0.014)	(123.590)
drug_procure	−0.015^***^	−128.365^**^
	(0.004)	(54.712)
pay_reform	−0.021^***^	−217.690^***^
	(0.006)	(72.433)
grassroot_cap	−0.009^**^	−95.228
	(0.004)	(59.766)
Controls	Yes	Yes
Province FE	Yes	Yes
Year FE	Yes	Yes
*N*	4,895	4,895
*R* ^2^	0.067	0.044

The control variables are consistent with those in Table 2, and the same applies below. ** indicates p < 0.05, and *** indicates p < 0.01.

### PSM-DID estimation

To address potential sample selection bias in the policy effect estimation, the PSM-DID method is employed, with 1:1 and 1:2 nearest-neighbor matching with replacement (Table 13). Across all matching specifications, the estimated coefficients of the Pooling treatment variable remain significantly positive for both inpatient service utilization and hospitalization costs, consistent with our baseline findings. These results confirm that the estimated policy effects are robust and are not driven by observable pre-treatment differences between the treatment and control groups.

**Table 13:** PSM-DID estimation results.

Matching strategy	Outcome	Pooling	*SE*	*p*-value	*R* ^2^	*N*
1:1 nearest neighbor with replacement	Inpatient service utilization	0.321	0.072	0.000	0.106	542
1:1 nearest neighbor with replacement	Hospitalization costs	472.413	125.332	0.000	0.085	737
1:2 nearest neighbor with replacement	Inpatient service utilization	0.248	0.062	0.000	0.097	542
1:2 nearest neighbor with replacement	Hospitalization costs	426.379	127.376	0.000	0.076	737

### Heterogeneity-robust estimator analysis

**Figure 5:**
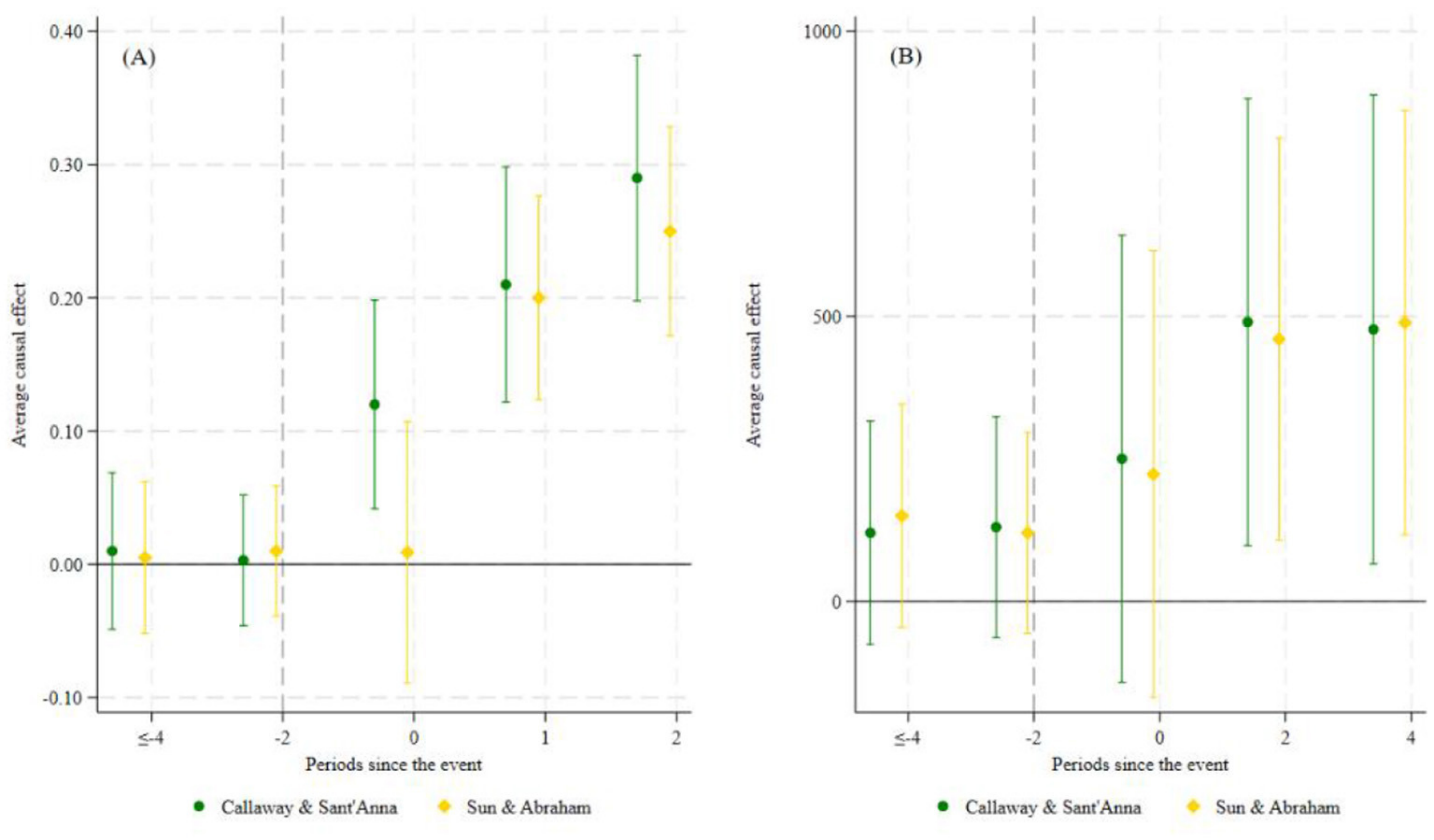
Heterogeneity-robust estimator analysis.

To address bias in conventional TWFE estimators in staggered DID with heterogeneous treatment effects, two robust estimators are employed: (47, CS) and (48, SA) (Figure 5). Panel (A) shows results for inpatient service utilization. Both estimators satisfy the parallel trends test, and yield significantly positive post-reform effect estimates. Panel (B) reports corresponding estimates for hospitalization costs, which similarly exhibit flat pre-trends and significant positive post-reform effects. Consistent patterns across these two estimators further confirm the robustness of our findings.

## Discussion

This study examines the impact of provincial-level pooling reform of the BMISURR on inpatient service utilization, hospitalization costs, and the distribution of these outcomes among insured individuals. The findings contribute to the literature by showing that provincial-level pooling is associated with higher inpatient utilization and hospitalization costs (13, 14), while also shaping important distributional patterns in inpatient care. The DID results indicate that provincial-level pooling significantly increases both inpatient service utilization and hospitalization costs. This finding is consistent with prior studies suggesting that a higher pooling level can expand effective insurance protection and stimulate healthcare use (36). When the pooling level is raised, the insurance fund expands, and the system's mutual-aid capacity is strengthened. In practice, this reform is often accompanied by improved reimbursement rates, broader benefit packages, higher payment ceilings, and more convenient cross-regional medical settlement (37). As a result, the effective price of inpatient care declines from the patient's perspective, releasing previously constrained demand and encouraging greater use of inpatient services. Meanwhile, expanded access to higher-level hospitals and more complex treatments may also increase average hospitalization costs. This mechanism can be illustrated by the experience of Hainan Province. After Hainan implemented unified provincial collection and expenditure of BMISURR funds in 2020, benefit levels were further improved: the reimbursement rate at tertiary designated institutions was raised to 65%, the number of covered chronic and special diseases increased from 25 to 40, and the annual maximum payment limit for resident medical insurance rose from 370,000 yuan to 450,000 yuan (38). These changes suggest that provincial-level pooling can improve benefit equalization and reduce administrative fragmentation. However, it may also stimulate greater utilization of inpatient services and higher expenditures when the expansion of insurance capacity is not fully matched by strict expenditure control and payment supervision.

Regarding pooling-model heterogeneity, the interaction-based DID results show that the provincial risk adjustment fund model contributes to a more substantial rise in both inpatient service utilization and hospitalization costs compared with the unified revenue-and-expenditure model. This disparity largely stems from differences in incentive mechanisms and governance structures. Under the unified revenue and expenditure model, funds are highly centralized at the provincial level, enabling provincial authorities to implement unified budgeting arrangement, expenditure supervision, and cost-control measures. By contrast, the provincial risk adjustment fund model allows local governments to retain substantial discretionary power over benefit expansion and expenditure management, while a portion of fiscal pressures is shared through the superior cross-regional adjustment pool (39–41). This arrangement loosens budget constraints, weakens the motivation for strict cost containment, and drives local governments to expand inpatient spending. Such spending expansion is mainly reflected in higher reimbursement rates, looser admission thresholds, and increased utilization of tertiary and high-level medical institutions. It is important to distinguish between the two types of estimates in this study, which target income-related distribution of these outcomes. Therefore, the decomposition results should not be interpreted as independent evidence proving that provincial-level pooling causally widens health inequality.

The concentration index results show that both inpatient service utilization and hospitalization costs remain concentrated among higher-income groups, a conclusion consistent with findings from existing literature (42). The decomposition results further indicate that pro-rich inequality in medical services is primarily shaped by structural factors rather than isolated policy arrangements. Across all decomposition specifications, income level acts as the most stable and leading positive contributor to pro-rich inequality, while healthcare resources also exerts a prominent influence. This evidence suggests that inpatient care inequality originates fundamentally from the interaction between socioeconomic stratification and imbalanced healthcare resource supply. The strong contribution of income suggests that even under a formally unified insurance scheme, individuals with higher economic status can better convert nominal insurance benefits into actual medical service access. Higher-income groups are more capable of affording hospitalization-related expenses, including deductibles, transportation fees, and caregiving expenditures, as well as other non-medical costs. Meanwhile, they generally possess superior health literacy, higher policy cognition, and stronger capabilities to optimize medical referral procedures and obtain treatment in higher-level hospitals. Therefore, the distributional effects of provincial-level pooling cannot be solely attributed to insurance system design. Instead, these effects are profoundly constrained by long-standing disparities in household economic capacity across different social groups.

The large contribution of healthcare resources further indicates that supply-side inequality serves as a key pathway sustaining pro-rich patterns in inpatient care. Hospitals and higher-level inpatient capacity are generally concentrated in economically advantaged regions. Accordingly, patients residing in these areas or with greater cross-regional mobility are more likely to gain access to advanced and intensive medical services. In this context, expanded insurance coverage does not automatically equalize healthcare access, as the geographical distribution of medical service supplies remains uneven. This finding explain why pro-rich inequality persists even after controlling for individual characteristics and policy variables. It also suggests that insurance reform implemented without corresponding adjustment in healthcare resource allocation tend to exacerbate disparities in actual medical access and hospitalization expenditure. The decomposition results also clarify why some contribution rates exceed 100%. Such numerical outcomes are statistically reasonable: specific variables drive significant positive effects on pro-rich inequality, whereas other covariates produce offsetting negative effects. In this study, variables such as health status partly offset the inequality generated by income gaps and imbalanced healthcare resources. Specifically, lower-income groups generally suffer from poorer health conditions, which increases medical demand in a pro-poor direction. Therefore, contribution rates above 100% should not be interpreted literally. These figures do not mean a single factor accounts for more than the total inequality; instead, they reflect that a given acts as the dominant positive contributor, while other determinants exert countervailing inhibitory effects. Collectively, these results suggest that provincial-level pooling is not the fundamental cause of inpatient care inequality.

On the contrary, decomposition results indicate that provincial-level pooling is positively associated with pro-rich inequality against a backdrop of pre-existing structural inequalities rooted in income stratification and uneven healthcare resource distribution. In essence, the reform enhances residents' medical purchasing power within an inherently unequal social and spatial healthcare system. Under such conditions, better-off individuals and residents of better-resourced regions disproportionately benefit from the expanded coverage of provincial-level pooling (43, 44). This pattern appears more pronounced under the risk adjustment fund model, which shows a stronger positive association with observed pro-rich inequality, especially in terms of hospitalization costs. The contrast between Hainan and Ningxia provides an institutional lens for understanding these differences. In Hainan, provincial-level pooling was implemented together with the “six unifications” framework. This reform integrated fund enlargement with centralized budgeting management, benefit standardization, and administrative coordination (45). By comparison, in Ningxia, although the resident insurance system was improved at the autonomous-region level, the policy architecture still combined region-wide pooling with graded administration and local implementation responsibilities, and adjustment funds were primarily deployed to address lower-level deficits (46). This difference is critical because the distributional consequences of pooling depend not only on the scale of consolidated funds but also on the authority of expenditure management and corresponding institutional constraints. Pooling reforms paired with centralized budget supervision and unified benefit governance are more conducive to balanced redistribution. In contrast, if pooling merely relies on intergovernmental fiscal adjustment without centralized expenditure regulation, reform dividends will be disproportionately captured by advantaged regions and high-income groups. For hospitalization costs, the pre-treatment event-study pattern is less stable than for inpatient utilization. Thus, causal inferences cannot rely solely on the parallel-trends figure alone. Instead, the robustness of core findings is verified by consistent results placebo tests, matching-based estimates, and heterogeneity-robust estimators all point in the same direction. Overall, the findings imply that improving the pooling level of BMISURR is necessary yet sufficient for advancing equity in inpatient care. Raising the pooling level can strengthen protection and boost medical service utilization. However, without targeted redistribution toward low-income groups, better integration with hierarchical diagnosis and treatment, and more balanced healthcare resource allocation, the structural roots of medical inequality will remain unresolved.

### Policy recommendations

Based on the above findings, several policy recommendations can be proposed accordingly. First, provincial-level pooling of BMISURR should move beyond formal unification of coverage and place greater emphasis on equity-oriented benefit design, with focused support for low-income households and groups with heavy medical burden. Given that income serves as the dominant factor to the observed pro-rich inequality in inpatient care, policy reform should directly target the economic barriers that prevent disadvantaged groups from converting nominal insurance coverage into accessible medical services. Provincial authorities may consider introducing differentiated deductibles and reimbursement rates for low-income enrollees, rural residents, and patients with severe or chronic conditions. Meanwhile, annual out-of-pocket expenditure caps should be implemented to relieve the financial pressure on households facing frequent or high-cost hospitalization. For patients who must seek inpatient care across counties or cities, especially those referred to higher-level hospitals, targeted referral subsidies covering transportation, accommodation, and caregiving costs can effectively lower non-medical barriers to care.

Second, provincial-level pooling reform should strengthen the ability of disadvantaged groups to use the entitled benefits. The decomposition results indicate that the observed inequality pattern is driven not only by reimbursement design, but also by differences in health literacy, policy awareness, and healthcare navigation capacity across socioeconomic groups. Therefore, in addition to benefit adjustment, provincial governments should develop health literacy improvement programs for the older adults, rural residents, and chronically disease patients. Practical measures include simplifying policy publicity materials, providing standardized reimbursement guidance via community and grassroots service stations, optimizing family-doctor-based referral mechanisms, and delivering case-management services for patients with long-term or high-cost hospitalization.

Third, reforms must address the unequal distribution of healthcare resources, which emerges as another major contributor to observed inpatient inequality. Exclusive reliance on provincial-level pooling fails to achieve equitable outcomes if high-quality inpatient services remain concentrated in better-resourced regions and tertiary hospitals. Therefore, provincial governments should combine pooling reform with stronger measures to improve the spatial equity of healthcare resource supply. Specific actions involves strengthening county-level inpatient service capacity, expanding the availability of key specialties and referral support in under-resourced areas, and introducing preferential payment incentives for hospitals and medical alliances serving less-developed regions. Provincial monitoring mechanism should be established to track whether pooled funds are disproportionately absorbed by tertiary hospitals in resource-rich areas and whether lower-level facilities are being crowded out.

Fourth, differentiated governance strategies should be formulated for distinct medical insurance pooling models, a conclusion validated by institutional practices observed in representative case provinces. Hainan, which shifted from the risk adjustment fund model to the unified revenue and expenditure model in 2020, provides an example of a more centralized reform pathway. According to its provincial policy on fund collection and expenditure management, Hainan established a province-wide institutional framework of “six unifications”, including unified fund revenue and expenditure management, unified budgeting, unified benefit policies, unified collection and disbursement procedures, unified responsibility-sharing arrangements, and a unified information system. Official policy documents indicate that following this reform, the reimbursement rate at tertiary designated medical institutions rose to 65%. The scope of covered chronic and special diseases expanded from 25 to 40, and the annual maximum payment limit for resident medical insurance increased from 370,000 yuan to 450,000 yuan. These tangible improvements suggest that when pooling is accompanied by a high degree of institutional centralization, the enlarged provincial fund pool is more likely to be translated into coordinated benefit equalization, stronger provincial oversight, and more consistent implementation across regions. By contrast, Ningxia illustrates the institutional operational logic of the risk adjustment fund model. Its 2019 policy on improving region-wide pooling of resident medical insurance established a management structure characterized by “region-wide pooling, hierarchical administration, and shared responsibilities.” Subsequent handling rules clarify that the autonomous region takes charge of policy formulation, supervision, adjustment fund allocation, and fiscal revenue and expenditure assessment. By comparison, municipal and county authorities agencies continued to undertake substantial responsibilities for benefit review, payment, settlement, local service management, and policy outreach. In addition, fiscal documents show that local governments must transfer a fixed proportion of resident insurance fund revenue to the autonomous-region adjustment fund. For instance, the 2022 remittance ratio for resident medical insurance was set at 7% of the previous year's actual fund revenue. Related policy explanations also indicate that the adjustment fund was primarily designed to compensate grassroots fiscal deficits. Supplementary regulations on budget management, performance evaluation, and fund supervision were introduced later to prevent unreasonable reliance on the provincial fiscal adjustment. These institutional features suggest that the adjustment-fund model mainly strengthens interregional deficit equalization rather than realizing full centralization of expenditure governance. Under such an arrangement, local governments may retain considerable discretionary power over benefit expansion and inpatient expenditure management, while externalizing partial fiscal burdens to the upper-level adjustment funds. This structure loosens budget constraints and creates conditions for unequal capture of the gains from pooling. This comparison explains the empirical finding that, relative to the unified revenue and expenditure model, the risk adjustment fund model generates a stronger expansion in medical spending and correlates more closely with pro-rich inequality in inpatient care. Therefore, future reform should not focus only on raising the formal pooling level. For provinces adopting the risk adjustment fund model, the provincial authority should move beyond deficit compensation and introduce more restrictive governance instruments. Key measures include equity-oriented earmarked grants for adjustment funds, performance-based allocation rules, expenditure-growth thresholds, and regular supervision to prevent excessive concentration of pooled resources on tertiary inpatient care or socioeconomically advantaged populations. Regions with persistently excessive hospitalization growth or high medical spending concentration should bear a greater share of fiscal responsibility. In contrast, areas with effective cost containment and equitable medical service provision can receive preferential adjustment fund support, so as to constrain unreasonable medical expenditure and narrow interregional health service disparities.

Finally, provincial-level pooling should be further integrated with hierarchical diagnosis and treatment systems and medical payment reform. If pooling merely improves financial accessibility without guiding patients to receive rational and hierarchical medical services, a large share of the reform benefits may continue to be occupied by groups with inherent advantages in accessing high-level hospitals. Therefore, county and prefectures-level governments should be encouraged to strengthen local inpatient service capacity and referral coordination. Multiple supporting measures can be implemented, including preferential reimbursement for appropriate county-level hospitalization, payment incentives for tightly integrated county medical alliances, standardized referral procedures connecting primary care, county hospitals, and provincial hospitals, and closer alignment between pooling reform and DRG/DIP payment mechanisms. In this way, provincial-level pooling can evolve from a single financing reform into a comprehensive institutional mechanism that promotes both sustainability and equity in inpatient care.

## Conclusion

Based on seven waves of the China Family Panel Studies from 2010 to 2022, this study examined the impact of provincial-level pooling reform of BMISURR on inpatient service utilization, hospitalization costs, and the observed distribution of these outcomes among insured individuals. Four main conclusions can be drawn. First, the DID results indicate that provincial-level pooling significantly increases inpatient service utilization and hospitalization costs among BMISURR enrollees. Second, the interaction-based heterogeneity analysis shows that both the unified revenue and expenditure model and the provincial risk adjustment fund model can effectively boost inpatient utilization and medical expenditure, and the risk adjustment fund model exerts a more prominent policy effect. Third, the concentration index results indicate that inpatient service utilization and hospitalization costs remain concentrated among high-income groups. Decomposition analysis further verifies that provincial-level pooling is positively associated with this observed inequality pattern. Fourth, both pooling models show positive associations with pro-rich inequality, and the provincial risk adjustment fund model presents a stronger correlation, especially regarding hospitalization costs.

## Limitations

This study has several limitations that deserve clarification. First, the latest CFPS data available for this analysis only covers the year 2022. A number of Chinese provinces have continued to advance provincial-level pooling reform after 2022. Therefore, the research conclusions are only applicable to the reform context from 2010 to 2022. Further research incorporating more recent waves and additional provinces will be needed to assess the longer-term effects and broader external validity of provincial-level pooling reform. Second, although this study adopts multiple empirical strategies to strengthen causal identification, including event-study analysis, placebo tests, PSM-DID, controls for concurrent healthcare reforms, and heterogeneity-robust estimators, residual endogeneity cannot be fully ruled out. In particular, time-varying unobserved factors—such as the popularization of new medical technologies, changes in hospital management practices, and demographic shifts represented by population aging, may interfere with inpatient utilization and hospitalization costs. Third, this study adopts two different analytical approaches with different inferential scopes. The DID models are designed to estimate the average effect of provincial-level pooling on inpatient service utilization and hospitalization costs, whereas the concentration index decomposition is used to describe how provincial pooling and other covariates are associated with the observed income-related distribution of these outcomes. Therefore, the equity-related decomposition results should be interpreted from the perspective of contribution correlation rather than regarded as independent causal estimates of inequality effects. Fourth, the research sample is restricted to BMISURR enrollees in provinces with comparable provincial-level-pooling arrangements. Hainan is excluded from the model heterogeneity analysis because of its mid-term institutional transformation of pooling models. For this reason, the research findings may not be directly generalizable to all provinces, regions with special institutional arrangements, or other medical insurance systems such as employee medical insurance. Finally, the data are based in part on self-reported survey responses, which may introduce recall bias and subjective measurement errors. This problem is particularly obvious for self-rated health status and retrospectively reported healthcare utilization and expenditures. Future studies could combine survey data with more objective administrative claims or hospital records to further improve measurement accuracy.

## References

[1] Yip W Fu H Chen AT Zhai T Jian W Xu R . 10 years of health-care reform in China: progress and gaps in universal health coverage. Lancet. (2019) 394:1192–204. doi: 10.1016/S0140-6736(19)32136-1 31571602

[2] Li YT Song Y. Current status, issues, and development trends of provincial pooling in China's basic medical insurance. Health Soft Sci. (2025) 39:75–80, 87.

[3] Zhang JH Zhang XM Wang SR Zhang Q Shuai XJ Wen HJ . Analysis of driving factors for provincial pooling in medical insurance: based on a geographically weighted regression model. China Health Insur. (2025) 2025:38–47. doi: 10.19546/j.issn.1674-3830.2025.4.005

[4] Liao CY Shi L. Study on the impact of provincial pooling of employee medical insurance on medical insurance fund expenditures and its mechanism. J. Jiangxi Univ. Financ. Econ. (2024) 2024:53–65.

[5] Li Z Zhang C. On the reform of individual financing mechanisms in resident medical insurance: from fixed amount to fixed proportion. China Health Policy Res. (2021) 14:1–10.

[6] Wang CQ. Who remains uninsured? A study on the demographic characteristics of participants in China's urban and rural resident medical insurance. Soc. Security Rev. (2023) 7:76–93.

[7] National Healthcare Security Administration of China. Statistical Bulletin on the Development of National Healthcare Security in 2023 (2024). Available online at: https://www.nhsa.gov.cn/art/2024/7/25/art_7_13340.html (Accessed November 26, 2024)

[8] Zeng W. Provincial pooling of basic medical insurance: experiences, dilemmas and countermeasures. Health Econ Res. (2024) 41:24–37.

[9] Xie M Yang J Yang Y. Provincial pooling of urban–rural residents' medical insurance, healthcare-seeking behavior and growth of medical expenditures. Chin J Health Policy. (2024) 17:10–8.

[10] Liu C Liu Z Nicholas S Wang J. Trends and determinants of catastrophic health expenditure in China 2010–2018: a national panel data analysis. BMC Health Serv Res. (2021) 21:526. doi: 10.1186/s12913-021-06533-x 34051762 PMC8164806

[11] Zhu X Mahal A Tang S McPake B. A Chinese conundrum: does higher insurance coverage for hospitalization reduce financial protection for the patients who most need it? Health Policy Plan. (2025) 40:287–99. doi: 10.1093/heapol/czae108 39520277 PMC11886816

[12] Wang H. Model choices for improving the pooling level of China's social medical insurance: a perspective based on international experience. Comp Econ Soc Syst. (2009) 2009:60–7.

[13] Li R Wu J Yang H. Impact of provincial pooling of employee medical insurance on medical expenditure: a study based on CFPS data. Insur Stud. (2022) 2022:83–98.

[14] Shen Y. Pooling level of medical insurance, utilization of medical services and health welfare: the mediating mechanism of medical expenditure increase under provincial pooling. Soc Secur Rev. (2022) 6:83–101.

[15] Deng K Ding Z Li J. Medical insurance and physician-induced demand in China: the case of hemorrhoid treatments. Int J Health Econ Manag. (2022) 22:257–94. doi: 10.1007/s10754-021-09318-1 34773531

[16] Sun J Yang YF Li HM. Performance analysis of enhancing pooling levels in employee medical insurance from a resource consumption perspective: a discussion on efficiency and equity. Chin Health Policy Res. (2024) 17:1–9.

[17] Liu H Zhao Z. Does health insurance matter? Evidence from China's urban resident basic medical insurance. J Comp Econ. (2014) 42:1007–20. doi: 10.1016/j.jce.2014.02.003

[18] Cheng L Liu H Zhang Y Shen K Zeng Y. The impact of health insurance on health outcomes and spending of the elderly: evidence from China's New Cooperative Medical Scheme. Health Econ. (2015) 24:672–91. doi: 10.1002/hec.3053 24777657 PMC4790431

[19] Wagstaff A Lindelow M. Can insurance increase financial risk? The curious case of health insurance in China. J Health Econ. (2008) 27:990–1005. doi: 10.1016/j.jhealeco.2008.02.002 18342963

[20] Li Y Li L Liu J. The efficient moral hazard effect of health insurance: evidence from the consolidation of urban and rural resident health insurance in China. Soc Sci Med. (2023) 324:115884. doi: 10.1016/j.socscimed.2023.115884 37018870

[21] Yuan H Han J Luo R. The moral dilemma of healthcare service utilization: a perspective from the consolidation of urban and rural resident health insurance policy in China. Health Econ Rev. (2025) 15:10. doi: 10.1186/s13561-025-00591-1 39964576 PMC11837384

[22] Bai L Gu H. Dilemmas and reflections on municipal pooling of basic medical insurance: based on a survey of five cities in Jiangsu Province. Health Econ Res. (2021) 38:35–8.

[23] Wang Z Yao J. The dilemma of medical insurance under the principal–agent framework: impact of provincial pooling on fund revenue and expenditure—evidence from urban employee basic medical insurance. Insur Stud. (2023) 2023:104–18.

[24] Yan X He S Webster C Yu M. Divergent distributions of physicians and healthcare beds in China: changing patterns, driving forces, and policy implications. Appl Geograp. (2022) 137:102626. doi: 10.1016/j.apgeog.2021.102626

[25] Pan J Shallcross D. Geographic distribution of hospital beds throughout China: a county-level analysis in 2011. Int J Equity Health. (2016) 15:179. doi: 10.1186/s12939-016-0467-9 27821181 PMC5100192

[26] Grossman M. On the concept of health capital and the demand for health. In: Determinants of Health: An Economic Perspective. New York: Columbia University Press (2017). p. 6–41. doi: 10.7312/gros17812-004

[27] Wagstaff A van Doorslaer E Watanabe N. On decomposing the causes of health sector inequalities with an application to malnutrition inequalities in Vietnam. J Econom. (2003) 112:207–23. doi: 10.1016/S0304-4076(02)00161-6

[28] Epo BN Baye FM. Decomposing poverty–inequality linkages of sources of deprivation by men-headed and women-headed households in Cameroon. J Econ Dev. (2016) 41:57–79. doi: 10.35866/caujed.2016.41.1.004

[29] Chirwa GC Suhrcke M Moreno-Serra R. Socioeconomic inequality in premiums for a community-based health insurance scheme in Rwanda. Health Policy Plan. (2021) 36:14–25. doi: 10.1093/heapol/czaa135 33263730

[30] Chen M Fang G Wang L Wang Z Zhao Y Si L. Who benefits from government healthcare subsidies? An assessment of the equity of healthcare benefits distribution in China. PLoS ONE. (2015) 10:e0119840. doi: 10.1371/journal.pone.0119840 25781163 PMC4362950

[31] Marfels C. Absolute and relative measures of concentration reconsidered. Kyklos. (1971) 24:753–66. doi: 10.1111/j.1467-6435.1971.tb00631.x

[32] Kjellsson G Gerdtham UG. On correcting the concentration index for binary variables. J Health Econ. (2013) 32:659–70. doi: 10.1016/j.jhealeco.2012.10.012 23522656

[33] Nanfu H Yajiao P Zhongqi L Hong S Hanfang Z Yuyu L . Influencing factors and equity of health status among the migrant elderly population in China. Chin J Gerontol. (2021) 41:4398–401.

[34] Li XR Zhang XM Ren Z Feng XW Gao X Song H. Evaluation of the equity in inpatient service utilization among migrant populations in eastern China. Med Soc. (2021) 34:12–5.

[35] Wagstaff A Paci P Van Doorslaer E. On the measurement of inequalities in health. Soc Sci Med. (1991) 33:545–57. doi: 10.1016/0277-9536(91)90212-U 1962226

[36] Dong B. The promotion of pooling level of basic medical insurance and participants' health: impact effects and mediating mechanisms. Int J Equity Health. (2023) 22:113. doi: 10.1186/s12939-023-01927-1 37287060 PMC10246363

[37] Zhu F. Policy choices for provincial pooling of basic medical insurance in China: based on international experience. China Med Insur. (2021) 2021:72–80.

[38] Wenchang City Medical Security Bureau Hainan Province. Hainan Province has Taken the Lead Nationwide in Implementing Provincial-Level Pooling of Medical Insurance Funds, Achieving Unified Collection and Disbursement with Positive Results (2025). Available online at: https://wenchang.hainan.gov.cn/wcsybj/ybjg/202103/0faf749bea014aac9dc51f64dfb65423.shtml (Accessed November 26, 2025).

[39] Zhou J Xing L Wang L . Impact of provincial pooling of medical insurance and quality of medical services in county-level medical institutions in Hainan Province on patients' healthcare-seeking behavior. Chin Hospitals. (2025) 29:22–6.

[40] Dong B. Current situation, problems and suggestions of provincial pooling of medical insurance: a policy text analysis. China Med Insur. (2023) 2023:30–6.

[41] Wu C. Study on equity issues in improving the pooling level of medical insurance. Med Philos (Ser A). (2015) 36:58–60, 93.

[42] Gu X Hui W. Universal medical insurance and equity in health service utilization: an analysis based on multiple waves of the China Family Panel Studies. J Soochow Univ (Philos Soc Sci Ed). (2024) 45:28–38.

[43] Wu J Liu Y Wang C Liu L Lu J. The effects of unified pooling arrangement on health inequity in China: a DID-RIF approach. BMC Health Serv Res. (2025) 25:145. doi: 10.1186/s12913-025-12304-9 39863904 PMC11762453

[44] Fu M Xu W. Influencing factors and experience models of provincial pooling of basic medical insurance in China. Cons Econ. (2019) 35:6–13.

[45] The People's Government of Hainan Province. Notice of the People's Government of Hainan Province on Issuing the “Interim Measures for Unified Collection and Distribution Management of Basic Medical Insurance Funds in Hainan Province” (2026). Available online at: https://www.hainan.gov.cn/hainan/szfwj/202001/90a4210879ff47f596bcb8451016866e.shtml (Accessed April 10, 2026)

[46] The People's Government of Ningxia Autonomous Region. Opinion of the People's Government of Ningxia Autonomous Region on Further Improving the Autonomous Region-wide Unified Management System for Basic Medical Insurance for Urban and Rural Residents (2026). Available online at: https://www.nx.gov.cn/zwgk/gfxwj/201911/t20191112_1845669.html (Accessed April 10, 2026)

[47] Callaway B Sant'Anna PHC. Difference-in-differences with multiple time periods. J Econom. (2021) 225:200–30. doi: 10.1016/j.jeconom.2020.12.001

[48] Sun L Abraham S. Estimating dynamic treatment effects in event studies with heterogeneous treatment effects. J Econ. (2021) 225:175–99. doi: 10.1016/j.jeconom.2020.09.006

